# Mixed corticomedullary tumor of the adrenal gland

**DOI:** 10.3389/fendo.2022.1026918

**Published:** 2022-09-16

**Authors:** Noriko Kimura, Teiich Motoyama, Jun Saito, Tetsuo Nishikawa

**Affiliations:** ^1^ Department of Clinical Research, National Hospital Organization Hakodate Hospital, Hakodate, Japan; ^2^ Department of Diagnostic Pathology, National Hospital Organization Hakodate Hospital, Hakodate, Japan; ^3^ Department of Pathology, Yamagata University School of Medicine, Yamagata, Japan; ^4^ Endocrinology and Diabetes Center, Yokohama Rosai Hospital, Yokohama, Japan

**Keywords:** mixed corticomedullary tumor, pheochromocytoma, adrenocortical tumor, ectopic ACTH, pathogenesis

## Abstract

Mixed corticomedullary tumor (MCMT) of the adrenal gland is an extremely rare tumor characterized by an admixture of steroidogenic cells and chromaffin cells in a single tumor mass simultaneously producing adrenocortical hormones and catecholamines; it is associated with ectopic adrenocorticotropic hormone (ACTH) in some cases. We reviewed and summarized clinicopathological data of 28 MCMTs, including four metastatic tumors in 26 previous reports. These reports included 21 females and 7 males, and the average tumor sizes were 4.8 ± 2.5 cm and 12.6 ± 6.4 cm in the non-metastatic and metastatic groups, respectively (P<0.001). The clinical manifestations and laboratory data were as follows: Cushing or subclinical Cushing syndrome, 58% (14/24); hypertension, 71% (17/24); elevated adrenocortical hormones, 75% (18/24); elevated catecholamines, 75% (18/24); and ectopic ACTH, 71% (10/14). All four patients with metastatic MCMTs had poor prognoses and elevated adrenocortical hormone levels; however, only two patients had elevated catecholamine levels. Immunohistochemistry was essential for the pathologic diagnosis of MCMTs. In this study, using an improved technique, we detected ectopic ACTH-producing cells in the same paraffin-embedded sections reported to be negative in our previous reports. As MCMT is composed of cells with embryologically different origins, its pathogenesis has been explained by various hypotheses. We compared MCMT to the adrenal gland of birds and the early stage of human fetuses, in which nests of chromaffin cells and steroidogenic cells admix without the formation of cortex and medulla. MCMT is characterized by the immaturity of organogenesis and might be classified as an adrenal embryonal tumor.

## Introduction

Mixed corticomedullary tumor (MCMT) of the adrenal gland is an extremely rare tumor characterized by an admixture of cell nests of both adrenal cortical and medullary cells in a single mass that produces adrenocortical steroid hormones and catecholamines (CA). MCMT also sometimes produces ectopic adrenocorticotropic hormone (ACTH) and can induce Cushing syndrome or subclinical Cushing syndrome. To date, less than 40 cases of MCMT have been reported since the first case was diagnosed in 1969. Herein, we reviewed 28 cases in 26 MCMT reports (1–26) and added some experimental data and hypotheses on the pathogenesis of MCMT.

Patients with MCMTs presented with varying levels of CA, adrenocortical hormones, and ACTH. Additionally, careful pathologic diagnosis is necessary to define adrenocortical or medullary cells using immunohistochemistry. All MCMTs previously reported were histologically confirmed. Although MCMTs were initially considered benign, 4 of the 28 patients with MCMT had distant metastases and poor prognoses. Thus, it should be considered that all MCMTs have some metastatic potential, as does pheochromocytoma (PCC).

Most MCMT studies focused on its pathogenesis because it is composed of different cell types: the medullary cells are derived from the neuro-ectodermal cells of the neural crest, and the adrenocortical cells are from the mesodermal layer, which is structurally similar to the adrenal gland. The most important difference between MCMT and the human adrenal gland is that the tumor cell nests of steroidogenic and medullary cells are randomly admixed in MCMT. In contrast, the human adrenal gland has distinct steroidogenic and medullary areas, namely the cortex and medulla. Many authors have attempted to explain the pathogenesis of this peculiar tumor through several hypotheses, including collision tumor, gene mutations, and stemness factors. Herein, we suggest perspective pathogenesis and ectopic ACTH in MCMT based on the comparative anatomy of the adrenal gland in vertebrates and human fetuses.

## Clinical manifestations and laboratory findings

We reviewed the characteristics of 26 previous studies involving 28 patients, including 24 patients with non-metastatic MCMTs from 22 studies (group A) (1–14, 16, 17, 19, 21, 22, 24, 26) and 4 patients with metastatic MCMTs from 4 other reports (group B) (15, 18, 20, 23).

The average patient age was 46.4 ± 12.3 years (mean ± standard deviation [SD], range: 25−66 years) in group A and 51.8 ± 26.4 years (range: 16–78 years) in group B. There were 21 female and 7 male patients, with a female:male ratio of 3:1; group A included 19 female and 5 male patients, while group B included 2 female and 2 male patients. The clinical manifestations and laboratory data were as follows: overall, 58% (14/24) had Cushing syndrome or subclinical Cushing syndrome, which was defined as autonomous glucocorticoid production without specific signs and symptoms of Cushing syndrome (27); 71% (17/24) had hypertension, and 75% (18/24) had elevated cortisol and CA levels. Plasma and/or urine CA, including epinephrine, norepinephrine, and dopamine, and their metabolites, such as metanephrine and vanillylmandelic acid, were measured in each institute. The types of elevated CA are as follows: epinephrine plus norepinephrine plus dopamine, one; epinephrine plus norepinephrine, five; metanephrine only, two; dopamine only, one; vanillylmandelic acid, three; and elevated CA with unknown types, six; and the analysis revealed that at least 44% (8/18 cases) were epinephrine-producing. The medullary component in four cases was unassociated with CA secretion (biochemically non-functional) and was identified histopathologically (12, 18, 20, 23). Further, adrenocortical cells produced cortisol in most cases and aldosterone in two cases, and only one case produced dehydroepiandrosterone sulfate (DHEA-sulfate). Seven of 12 previous cases with elevated cortisol had unsuppressed ACTH levels, despite a hypercortisolemic state (19). The detectable ACTH level was not grossly elevated, as is usually seen from pituitary or ectopic sources (19). We added six subsequent cases from the reports after Lwin et al. (19) and found that 71% (10/14) of MCMTs had subclinical Cushing syndrome with ectopic ACTH. In cases with Cushing syndrome, ectopic ACTH syndrome was defined as ACTH dependent when the plasma ACTH level was >15 pg/ml (reference range: 10–50 pg/ml) and ACTH independent when the plasma ACTH level was <5 pg/ml based on a previous report (23). Among the eight ACTH cases examined with immunohistochemistry (2, 3, 9, 19, 22–24, 26), only three cases showed focal positivity for ACTH (2, 23, 26).

## Pathology of MCMTs

MCMTs were first defined histologically as cortical cells with round, regularly shaped, and rather small nuclei, and medullary cells with basophilic cytoplasm and nuclei with more varied appearance (1). The adrenocortical and medullary components’ ratios may differ between cases. In most cases, the cortical cells have mild abnormality; however, some metastatic MCMTs had cortical cells with malignant features described as Weiss’s criteria 7 (20). Both cells of the medullary and cortical components in MCMT are apparently tumor cells similar to PCC and adrenocortical neoplasm morphology and biology. MCMT is a very peculiar tumor, and its histology may vary from case to case, especially if it is metastatic.

The mean ± SD tumor sizes of non-metastatic and metastatic MCMTs were 4.8 ± 2.5 cm (range: 2.5–11.8 cm) and 12.6 ± 6.4 cm (range: 8–22 cm), respectively. Metastatic MCMTs were larger than non-metastatic MCMTs (P<0.001). In non-metastatic MCMTs, the cortical cells were usually uniformly shaped with mild nuclear atypia and had cortical adenoma features. Meanwhile, the medullary cells had slightly basophilic cytoplasm with hyperchromatic, irregularly shaped nuclei and nucleoli arranged in a zellballen pattern, which are compatible with those of PCC. Thus, MCMT was considered a mixed tumor of cortical adenoma/carcinoma and PCC.

### Immunohistochemistry

Immunohistochemistry was performed to distinguish the medullary cells from the cortical cells in the MCMTs. Chromogranin-A (CgA) and synaptophysin (SP) were the most frequently used antibodies to identify the medullary cells in 25 and 10 tumors, respectively. In addition, catecholamine synthesizing enzymes, such as tyrosine hydroxylase (TH), dopamine-b-hydroxylase (DBH), and phenylethanolamine-N-methyltransferase (PNMT), were positive in four cases (2, 9, 22, 26). Insulinoma-associated protein 1 (INSM1) was detected in one (22) case with two recently reported cases after our review (2, 9). CgA and SP were the most frequently used antibodies for confirming neuroendocrine tumors, including PCC. However, both antibodies and INSM1 are also markers for epithelial neuroendocrine tumors (eNETs) and are not specific markers for PCC. The combined use of these antibodies and TH or DBH is suitable for detecting CA-producing cells (28). The adrenocortical component was confirmed by inhibin-alpha in 11 cases, steroidogenic factor 1 (SF-1) in six cases, calretinin in four cases, and melan-A in four cases. Steroidogenic enzymes (3β-hydroxysteroid dehydrogenase; 11 β-hydroxylase [CYP11β1], p450c21, and p450c17) were positive in four cases. Among these antibodies for adrenocortical cells, SF-1 is presently the most universally used antibody to identify adrenocortical cells. The analysis of hormone products showed that the cortical cells and medullary cells were compatible with those present in adrenocortical adenoma and PCC, respectively. Electron microscopic examination was used to identify neuroendocrine granules in the cytoplasm of medullary cells and mitochondria with tubulovesicular cristae for adrenocortical cells (2, 4, 5). Sudan III stain was used for fat globules in adrenocortical cells (2, 3).

**Figure 1:**
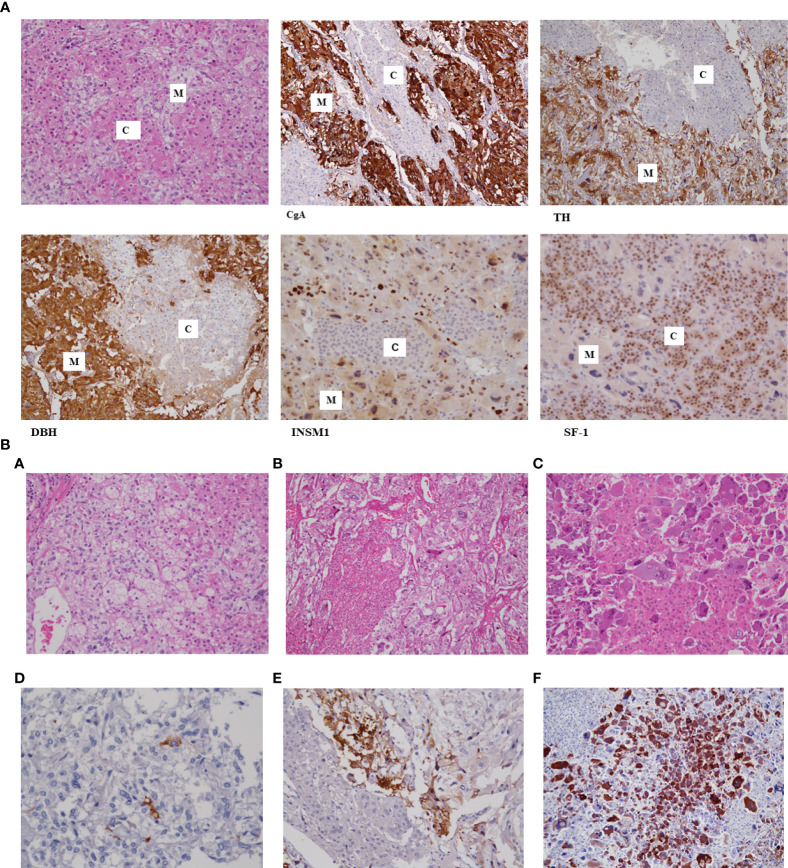
**(A)** Histology of mixed corticomedullary tumor (MCMT) with immunohistochemistry. The tumor is composed of nests of cortical cells with eosinophilic cytoplasm and round nuclei **(C)** and medullary cells with pale cytoplasm and hyperchromatic, irregularly shaped nuclei **(M)**. Cell nests of medullary cells are positive for chromogranin-A (CgA), tyrosine hydroxylase (TH), dopamine beta-hydroxylase (DBH), and INSM1; however, cortical cells are negative for all these biological markers but positive for SF1. **(B)** Histology of three cases of MCMTs with subclinical Cushing syndrome. Three cases of MCMT are demonstrated. The upper and lower lines are the same cases subjected to hematoxylin-eosin and adrenocorticotropic hormone (ACTH) immunohistochemical staining, respectively. Tumor cells in the left line show mild atypia in both cortical cells and medullary cells **(A)**, and there are few ACTH-positive cells **(D)**. Nests of medullary cells in the middle line show small and large irregular nuclei **(B)** and stain positively for ACTH in the adjacent area of cortical cells **(E)**. Medullary cells in the right line have irregularly shaped large basophilic cytoplasm and hyperchromatic large nuclei, especially those adjacent to eosinophilic cortical cells **(C)**, and these medullary cell components strongly demonstrate ACTH immunoreactivity **(F)**. Cortical cells in these cases show very mild atypia and are compatible with cortical adenoma.

Given that Cases # 2, 3, and 9 were our own previously reported cases, we re-examined the immunohistochemical staining of ACTH using novel modalities, including an autoimmunostainer for the same buffered formalin-fixed, paraffin-embedded blocks used before. Briefly, the immunohistochemical procedures for ACTH were performed using an automated immunostainer (Benchmark, Ventana, Tucson, AZ, USA) according to the manufacturer’s protocol. The primary antibody for ACTH was mouse monoclonal, clone 02A3 (DAKO), and the final dilution was 1:100. We did not need any enhancement for materials, and the incubation time was 32 minutes. The positive control was a human pituitary gland, and the negative control was phosphate-buffered saline. There was no nonspecific ACTH staining. The results revealed that all three MCMTs clearly demonstrated cell nests of medullary cells positive for ACTH immunoreactivity (**Figure 1**). This suggested that some medullary cells of MCMT with subclinical Cushing syndrome could produce ectopic ACTH. These ectopic ACTH-producing tumor cells were characteristically bizarre cells with abundant basophilic cytoplasm and irregularly shaped, large nuclei located at the intersection between the nests of cortical cells and smaller medullary cells. The smaller medullary cells with mild atypia did not show ACTH immunoreactivity. It is speculated that morphologically atypical medullary cells with bizarre nuclei have some genetic changes that produce both catecholamines and ACTH simultaneously, as shown in ectopic ACTH-producing PCC (29). The lack of ACTH reactivity in previous reports with subclinical Cushing syndrome may be due to the low levels of ACTH produced by the tumor cells and the lower sensitivity of immunohistochemistry at the time of the study, resulting in false negative staining as suspected by Lwin et al. (19). The reasons of discrepancy between our results of ACTH and the original data are considered as following; Immunohistochemical principle is basically same as before, however, recent progress in immunohistochemistry using an autoimmunostainer contributed to get more sensitive and specific results compared to the years of the original technique was performed. Furthermore, immunohistochemistry is now daily used technique in pathology laboratories but not a special technique for research as used before. Both progress of instruments and human techniques may be the reasons for detecting ACTH this time. However, in the above three cases, medullary cells with mild atypia had very few cells immunoreactive to ACTH, suggesting that the grade of histological atypia of medullary cells is related to the ectopic ACTH production. The immunohistochemical data are summarized in **Table 1**.

**Table 1:** Immunohistochemical markers used for identifying medullary cells and cortical cells in 28 previous reports of mixed corticomedullary tumors of the adrenal gland.

Antibodies	Medullary cells (Cases)	Cortical cells (Cases)
Chromogranin A	25	0
Synaptophysin	10	0
S100 for sustentacular cells	3	0
Catecholamine synthesizing enzymes: TH, DBH, PNMT	4	0
INSM1	3	0
ACTH for subclinical Cushing syndrome	4	0
Inhibin alpha	0	11
SF-1	0	6
Melan A	0	4
Calretinin	0	4
Steroid hormone synthesizing enzymes (3β-HSD, P450c21, P450c11, P450c17)	0	4
Pancytokeratin	0	5

TH, tyrosine hydroxylase; DBH, dopamine-b-hydroxylase; PNMT, phenylethanolamine N-methyltransferase; INSM1, insulinoma-associated protein 1; ACTH, adrenocorticotropin; 3b-HSD, 3 beta-hydroxysteroid dehydrogenase; P450c21, 21beta hydroxylase; P450c11, 11beta-hydroxylase; P450c17, 17alpha-hydroxylase.

### Other concurrent lesions

The previously reported MCMTs had other concurrent lesions, such as neurofibromatosis type 1 (14), myelolipoma (6, 9, 13), aldosterone-producing adrenocortical micronodules (26), and spindle cell sarcoma (4).

## Metastatic MCMT

Distant metastasis was observed in four patients (15, 18, 20, 23). The metastatic sites were the liver in four patients, lung in two patients, and posterior stomach in one patient. One patient had local recurrence in addition to liver metastasis. All patients with metastasis had elevated steroid hormones (three patients with cortisol and one patient with DHEA-sulfate); however, two patients had no elevated catecholamines (20, 23). One patient only had elevated cortisol levels at the time of primary tumor diagnosis, following which the CA levels were elevated at the time of liver metastasis (18). Only one patient had both elevated CA and steroid hormone levels from the time of primary diagnosis (15). Metastatic MCMTs produced more adrenocortical hormones than CA.

Histologically, all MCMTs with metastasis had both components of cortical tumor and PCC. The histological description of the metastatic MCMTs only included information about the primary tumors without any information about the metastatic sites. One case only described the medullary component without describing the cortical component; the tumor cells had marked nuclear pleomorphism, abundant mitotic cells with atypical mitotic figures, confluent geographic tumor necrosis, and vascular invasion in the PCC component (15). Another case was described as a high-grade undifferentiated carcinoma with evident tumor necrosis and focal vascular invasion. Many of the cells were large and pleomorphic, and some were multinucleated and bizarre with frequent mitoses up to 4/high-power field with aberrant forms. A Weiss score 7 was given, indicating adrenocortical carcinoma; however, no description of the medullary component was provided (20). From these descriptions, metastatic MCMTs had features of adrenocortical carcinoma or high-grade PCC (30).

These patients had poor prognoses due to highly progressive tumors. Two patients died 18–24 months postoperatively (18, 20), while two other patients deteriorated with metastatic tumors postoperatively (15, 23).

## Discussion

### Terminology of MCMT with metastasis

The terms “mixed corticomedullary carcinoma” were used for three cases (15, 18, 20) and “malignant mixed corticomedullary tumor” for the other case (23). The term “corticomedullary” carcinoma provides the impression that metastasis occurs only in the cortical component and not in the medullary component. All four MCMTs with metastasis had histological components of both cortical and medullary cells; however, biologically, all had elevated steroid hormones, while two cases had unelevated CA. The term “mixed corticomedullary carcinoma” seems to be inappropriate because the medullary component is non-epithelial; instead, it should be called sarcoma when it is pathologically diagnosed as malignant. The World Health Organization Endocrine Tumor Classification 4th edition (2017) recommends using metastatic pheochromocytoma and avoiding the terms “benign” or “malignant” to classify PCC. We would like to suggest the term “metastatic MCMT” instead of “mixed corticomedullary carcinoma” or “malignant MCMT.” There were only four metastatic MCMTs in the previous reports; however, patient follow-up time was insufficient, especially in the recently reported cases, and life-long follow-up may be necessary as requested for patients with PCC.

### Pathogenesis of MCMT

While previous reports have focused on the pathogenesis of MCMT, it remains unclear. The separate embryological origin of the adrenal medulla and cortex was previously suggested to favor a two clonal collision tumor (4, 5, 18). Disruption of the normal cortical-chromaffin cell interactions (paracrine interactions) by unknown mechanisms could theoretically result in trophic stimulation of both cell lineages and provide a possible explanation for the development of MCMTs (7, 14). Genetic testing was negative for *RET, VHL, SDHB, SDHC*, and *SDHD* (8). We detected immunohistochemical reactivity of *SDHB* in MCMTs, which ruled out *SDHx* gene mutations (data not shown). Only one case had NF1 (14), but majority of the tumor component of this patient was a composite PCC, and only 20% was cortical. A whole exome sequencing analysis using genomic DNA extracted from peripheral leukocytes and paraffin-embedded tumor tissue revealed no germline or somatic gene alterations (22), such as in *PRKACA, CTNNB1, GNAS, ARMC5, PRKAR1A, PDE11A*, or *PDE8B*, which have been reported in adrenal cortisol-producing adenomas (31). Moreover, no germline or somatic gene mutations were found in genes that have been reported in PCC, such as *NF1, RET, VHL, SDHx, TMEM127, MAX, HIF2A, PHD1/PHD2, FH, KIF1B, DNMT3A, IDH1*, or *SLC25A11* (32). Among the 10 genes detected as possible pathogenic candidates, Kanzawa et al. (22) focused on fibroblast growth factor receptor 4 (*FGFR4*). A homozygous *FGFR4-G388R* germline variant was identified in MCMT, which was suggested to influence the development of the adrenocortical component but not the PCC component.

Another hypothesis is based on a genetic event in stem cells which gives rise to cells constituting the cortex and the medulla. Immunohistochemistry using various tumor stem cell-specific markers, including acetaldehyde dehydrogenase 1, CD44, CD133, Nestin, NGFR, and SOX9, revealed short spindle-positive cells scattered within the tumor, suggesting the involvement of tumor stem cells (21). Double-labeling immunohistochemistry identified the presence of a few spindle cells within the tumor immunoreactive for both cortical and medullary antigens (23). Along with the positive immunofluorescence for cancer stem cell biomarkers (OCT4, NANOG, and SOX-2), these findings indicated the involvement of primitive embryonic cells as the origin of MCMT and concluded that MCMT may not come from colliding tumors, but from a single stem cell. Chiou et al. (24) reported that the previously well-known mutations for adrenocortical adenoma (*GNAS, CTNNB1, PRKAR1A, PRKACA, PDE11A, PDE8B, KCNJ5, CACNA1D*) (31) and PCC (*RET, VHL, NF1, SDHA, SDHB, SDHC, SDHD, SDHAF2, TMEM127, MAX, EGLN1(PHD2), EPAS1(HIF2A), KIF1B, MET, FH*, and *H-RAS*) (32) were not detected in their MCMT. However, immunohistochemistry confirmed that the stemness markers SOX2, CD44, and OCT4 were highly expressed in MCMT with greater adrenocortical adenoma density than in PCC. Several mutations were also identified in membrane receptors, such as *LRP5* p.C1548F, *IGFBP2* p. L21insPLL, *GPR39* p.V230A, *EMR2* p.S523F, which may be associated with MCMT development (24). Stemness activation may drive tumor formation, and the complex proliferative signaling caused by germline and somatic mutations may accelerate tumor growth. The mechanism by which stemness and asymmetrical tumorigenesis in the adrenal gland of MCMT are initiated remains unclear (24). Although cancer stem cells have been observed in MCMT as well as PCCs and paragangliomas (33) but also in other endocrine, non-endocrine, and neural tumors. The precise role of cancer stem cells and mutations in membrane receptors in MCMT tumor formation remains further accumulation of experiments.

### MCMT and the bird/fetal adrenal gland

Apart from cancer stem cell analysis, we would like to suggest a different approach to investigate MCMT tumorigenesis based on comparative endocrinology. MCMTs are histologically similar to the adrenal gland of birds, in which the catecholaminergic tissue is dispersed in the corticosteroidogenic tissue (34). A comparative endocrinology study showed evidence of considerable interspersion of the two components of chromaffin cells and corticosteroidogenic tissues in birds and most amphibians. However, the cortex-medulla relationship in most mammals represents a division into two separate tissues through contiguous components (35).

In humans, the fetal adrenal glands are detectable around the 6^th^ week of development, and its morphology is completed around the 7^th^ gestational week (35). The morphological and steroidogenic functions of the fetal adrenal cortex are formed around the 7^th^ gestational week (36). The neural crest-derived chromaffin cells stain positively for CgA and migrate toward the adrenal cortex as islands in the 6^th^–7^th^ gestational weeks. These chromaffin cells initially appear as small clusters or nests scattered throughout the cortex, where they gradually invade the medial aspect of the cortical tissue along the central vein at the 7^th^–12^th^ gestational weeks to gain a central position and then form around the adrenal medulla (36). However, the enzymes involved in CA biosynthesis can be detected as early as the 6^th^ gestational week (37, 38). Therefore, the adrenal glands of birds and human fetuses at the 6^th^–7^th^ gestational weeks are similar, forming an independent organ with admixed corticomedullary cell nests that produce steroid hormones and CAs. MCMT is characterized by the immaturity of organogenesis and might be classified as an embryonal tumor of the adrenal gland.

### ACTH production in MCMT

Small amounts of ACTH are released within the adrenal gland during splanchnic nerve stimulation in the functionally hypophysectomized calf (39). The epithelial hypophysis has long been believed to originate from Rathke’s pouch (RP). However, experiments on some amphibian species showed that the primordium of the epithelial hypophysis originates from the anterior neural ridge and migrates underneath the brain to form an RP-like structure (40). Further, early rat embryo culture confirmed that adenohypophyseal cells originate from the rostral end of the neural plate before RP formation (41). These animal models suggest that the anterior pituitary gland also exists close to the neural crest in human fetuses. It could be hypothesized that some anterior pituitary cells may migrate into the neural crest, become part of chromaffin cell nests, and produce ACTH during medullary component formation in early fetal stages as MCMT. However, further investigations are necessary to confirm this hypothesis.

## Data availability statement

The datasets presented in this article are not readily available because only immunohistochemical data are added in this manuscript. Requests to access the datasets should be directed to kimura.noriko.sf@mail.hosp.go.jp.

## Ethics statement

This study was approved by the institutional review board of the National Hospital Organization Hakodate Hospital (#R4-0730001).

## Author contributions

NK and TM contributed to the pathology research, and JS and TN contributed to the clinical research. NK re-examined the immunohistochemistry of ACTH, SF-1, INSM1, TH, DBH using the same paraffin-embedded blocks of the tumors that were used for the original manuscripts of the above references #2, 3, and 9, and confirmed that all those tumors contained cells immunoreactive to ACTH and the other antibodies. NK wrote the manuscript text and prepared **Figure 1** and **Table 1**. All authors reviewed and approved the manuscript.

## Acknowledgments

We are grateful to Professor Sakae Kikuyama, Waseda University, Tokyo, Japan, for excellent suggestions for the development of endocrine organs.

## Conflict of interest

The authors declare that the research was conducted in the absence of any commercial or financial relationships that could be construed as a potential conflict of interest.

## Publisher’s note

All claims expressed in this article are solely those of the authors and do not necessarily represent those of their affiliated organizations, or those of the publisher, the editors and the reviewers. Any product that may be evaluated in this article, or claim that may be made by its manufacturer, is not guaranteed or endorsed by the publisher.
